# COVID-19 in Older Adults at the Time of the Omicron Variant

**DOI:** 10.3390/jcm11185273

**Published:** 2022-09-07

**Authors:** Maurizio Gabrielli

**Affiliations:** Department of Emergency, Fondazione Policlinico Universitario Agostino Gemelli IRCCS, Università Cattolica del Sacro Cuore, Largo Gemelli 8, 00168 Rome, Italy; maurizio.gabrielli@policlinicogemelli.it; Tel.: +39-06-30157014

Since its outbreak, COVID-19 has had a significant impact on older adults worldwide. In fact, either morbidity or mortality, often secondary to acute pulmonary failure, extrapulmonary manifestations, or sequalae, better known as long COVID, are significantly more common in the elderly [1,2,3]. They are at a high risk of poor prognosis, especially when age is associated with frailty and multimorbidity [1,4].

This worse outcome may be due to several factors that often influence each other: an age-related global dysfunction of the immune system, to describe which the terms “immunosenescence” and “inflammaging” have been created; the higher prevalence of diseases and medications, leading to immunosuppression; and a significantly greater prevalence of comorbidities such as diabetes mellitus, cardiovascular, pulmonary and renal disorders, and other chronic diseases [2,3,5,6].

To date, however, much has changed since the start of the pandemic regarding the clinical features and outcomes of SARS-CoV2 infection, especially in the elderly. A complex combination of several factors has contributed to this change across the multiple waves of the pandemic. A better understanding of the pathophysiology and clinical course of COVID-19, the growing and widespread knowledge of non-invasive respiratory support techniques, and the introduction of effective pharmacological treatments have led to the better management of these patients. Nevertheless, these measures will not be enough to prevent the death toll from increasing further. Two main reasons, acting in synergy, have completely changed the clinical scenario of COVID-19. First, the availability and large-scale administration of several specific vaccines that have been found to be substantially safe and effective. Second, the appearance of new variants: Delta before, and Omicron after. The latter, with its two sub-variants (BA.1 and BA.2) dividing the globe, now undisputedly dominates new infections worldwide [7].

Several points in both arguments deserve a more detailed analysis, especially regarding the elderly population and the current predominant variant.

In December 2020, the availability of vaccines to protect against the spike protein of SARS-CoV2 dramatically changed the outlook on the COVID-19 pandemic. All approved vaccines were administered to deal with the first variants of the virus and repeated doses were required to effectively protect against infection and worse clinical outcome, and to protect the elderly in particular [8]. However, the significant and rapid loss of protection against infection, symptomatic infection, hospitalization, severe/critical infection and mortality made it essential to administer booster doses [9]. Unfortunately, at present, only the most developed countries have achieved the goal of mass vaccination. It is well-known that the appearance of the last two variants, which emerged during a new wave of the pandemic following the introduction of the vaccines, changed the scenario again.

The Delta variant (B.1.617.2 lineage) alarmed health systems around the world because it combined superior infectivity to previous variants with poor clinical outcomes [10,11]. Fortunately, vaccination, especially after the booster dose, while not having a great effect on the mitigation of viral transmission, had a significant impact on morbidity and mortality, particularly in older adults with multimorbidity [11,12].

The Omicron variant (B.1.1.529 lineage) was identified for the first time in South Africa in November 2021. A few weeks later, the World Health Organization declared it a variant of concern [13]. Between December 2021 and January 2022, Omicron spread rapidly worldwide, becoming today the cause of >99% of new infections. The high transmissibility (Omicron is three times more transmissible than Delta) is clearly the cause of its rapid spread in several countries, even in the presence of a complete vaccination course and previous infection with other variants [10]. The genome of B.1.1.529 lineage has more than 55 mutations, most of them related to the spike protein. The substantial modification of the characteristics of this protein may enhance viral fitness and enable antibody evasion [14]. In their refined in vitro research on the serum of patients infected by the B.1.1.529 lineage, Planas et al. demonstrated that Omicron escapes vaccine-induced antibodies and is not neutralized by sera from convalescent COVID-19 patients collected ≥6 months after clinical recovery [14]. On other hand, Omicron is neutralized by antibodies generated by a booster vaccine dose or by the vaccination of previously infected individuals. However, the titers of neutralizing antibodies against Omicron are much lower than those against Delta [14]. These results agree with the findings of several clinical studies, all confirming the further increased transmissibility of the actual prevalent variant. A meta-analysis by Madewell showed that household secondary attack rate was higher for Omicron than for Delta: 42.7% (95% CI, 35.4% to 50.4%) with respect to 29.7% (95% CI, 23.0% to 37.3%) [15]. The total vaccine effectiveness on infection was 64.4% (95% CI, 58.0% to 69.8%) for Delta and 35.8% (95% CI, 13.0% to 52.6%) for Omicron [15]. Receiving three doses of an mRNA vaccine was associated with significantly higher protection against symptomatic infection by the Omicron variant than being unvaccinated or receiving two doses. However, the higher odds ratios (OR) for Omicron shown in the study by Accorsi et al. are indicative of less protection for Omicron than for Delta [16]. The results are similar for patients of an older age [17].

A few studies have assessed the effect of a fourth dose on the transmissibility of B.1.1.529 lineage, mostly in patients ≥60 years of age. Compared to the third dose, the additional vaccine booster provides additional protection, but this result is weak and transient, as it peaks 2–3 weeks after administration and drops dramatically after about 10 weeks [18,19].

Fortunately, according to the literature data, Omicron is associated with a better clinical outcome than Delta. The likelihood of a visit to the emergency room, admission to hospital, intensive care unit admission, and the need for mechanical ventilation was significantly lower for Omicron [20,21,22]. Furthermore, vaccines have strong protection against severe illness from this variant. Booster vaccination with an mRNA vaccine was highly protective against hospitalization and death in Omicron cases, which was not affected by the vaccine used for the first two doses [20,23]. Even in the Omicron era, older age, frailty and multimorbidity remain significant risk factors for a worse clinical outcome [24]. The good news is that booster vaccination also significantly improves the clinical outcome of older adults, even more if they are frail and have comorbidities [17,24].

Administration of a fourth dose of the mRNA vaccine was associated with a further significant improvement in the clinical outcome, ensuring good protection against severe forms of COVID-19 [18,19,25,26]. Most of these studies enrolled subjects ≥60 years of age, and the results were confirmed in patients with more advanced age, frailty and multimorbidity [25,26]. Among patients with a mean age of 80 years admitted to hospital for symptoms of SARS-CoV2 infection, recent fourth dose administration was associated with significant protection against mechanical ventilation or death, compared to three doses (OR 0.51; 95% CI 0.3–0.87) [25]. In a large study of over 60,000 long-term care facility (LTCF) residents aged ≥60 years, vaccine effectiveness, compared with unvaccinated, increased with each additional dose. The effectiveness of the fourth dose against severe outcomes was 86%, with a marginal effect of up to 40% compared to the third dose [26]. These results are similar to those of another large prospective study of LTCF residents ≥60 years of age in Israel [27].

To date, little is known about Omicron’s response to monoclonal and antiviral drugs, which can be used in the treatment of COVID-19 in the elderly at different times in the natural history of symptomatic infection. Monoclonal antibodies, alone or in combination, act against the SARS-CoV-2 spike protein. They are indicated for adult patients at high risk of progression to severe disease, and, as is well known, age is one of the most important risk factors. Planas et al. showed that the Omicron variant was completely or partially resistant to neutralization by nine different monoclonal antibodies [14]. In a similar study, Takashima et al. showed that only some monoclonal antibodies (casirivimab, tixagevimab, cilgavimab, sotrovimab) or one combination (tixagevimab–cilgavimab) had neutralizing ability against the serum of a patient infected by Omicron. However, this susceptibility was partial and extremely lower than that observed against previous variants, including Delta [28].

In the same study, the authors tested the effectiveness of three different antiviral molecules: remdesivir, molnupiravir, and PF-07304814. All three showed similar efficacy to the previous strains [28]. These findings on antivirals appear to be confirmed by clinical studies carried out on older adults. In patients infected with Omicron, nirmatrelvir significantly reduced hospitalization and death from COVID-19 only in subjects ≥65 years of age and not in younger ones [29]. Among patients with a median age of 80 years admitted to hospital for severe COVID-19 from Omicron variant, remdesivir was associated with protection against mechanical ventilation or death (OR 0.65; 95% CI 0.44–0.96) [25].

Finally, according to the National Institute for Health and Care Excellence, Long COVID is defined as the presence of new or ongoing symptoms ≥4 weeks after the start of acute symptoms of SARS-CoV2 infection. Again, age proved to be an independent risk factor for sequelae, and vaccination of previously infected patients was associated with the relief of symptoms of long COVID, at least in the median follow-up of about 2 months [30,31]. Long COVID was significantly less prevalent after Omicron than after Delta infection (4.5% versus 10.8%, OR ranging from 0.24 to 0.50 accordingly with the vaccine status). These results were also confirmed after stratification by age group [32].

In conclusion, the available literature on the clinical outcome of older adults with COVID-19 and the effects of vaccination on them seems to reassure that, to date, the B.1.1.529 lineage is probably the best variant that could be expected. Indeed, the Omicron variant is associated with reduced disease severity, and full vaccination, even more after the fourth dose, offers strong protection against severe/critical COVID-19. However, it is not well known how long the efficacy of repeated doses remains valid on these endpoints, and therefore how often to repeat the vaccination. This is extremely important in the elderly, in whom, as already mentioned, the immune system is less robust or even dysfunctional. Another problem that remains is the high transmissibility of Omicron, which the available vaccines seem to effect only slightly and transiently. The third and fourth doses seem to do better, but the results remain unsatisfactory. The reduced effectiveness of current vaccines in preventing Omicron infection have underlined the urgent need to develop new ones in line with viral evolution. Higher Omicron neutralizing antibody titers have been observed with Omicron-containing mRNA vaccines compared to the Prototype one [33]. These preliminary but encouraging results have recently prompted several national pharmaceutical regulatory agencies to approve the Moderna and Pfizer-BioNTech bivalent COVID-19 vaccines for use as a booster dose. It will be interesting to verify on large samples, in different age groups, and with longer follow-up, the efficacy and safety of these new vaccines.

Finally, all the mutations involving the spike protein seem to significantly reduce the effectiveness of several monoclonal antibodies, which, in the case of the previous variants, have been shown to be effective in preventing the progression of COVID-19 towards severe forms. Obviously, this could mean the loss of an excellent preventative measure for older adults. However, in this regard, there are a lack of clinical trials that are equally urgent and aimed at verifying the results of in vitro studies. If confirmed, these data would suggest developing new monoclonal antibodies targeting the Omicron variant.

I hope that these insights can stimulate valuable contributions from other authors to be shared with the international scientific community.
